# Digital Image Correlation-Based Bolt Preload Monitoring

**DOI:** 10.3390/s26030913

**Published:** 2026-01-30

**Authors:** Linsheng Huo, Liukun Zhao, Aocheng Hu, Fanwei Meng, Hongnan Li

**Affiliations:** State Key Laboratory of Coastal and Offshore Engineering, Dalian University of Technology, Dalian 116024, China; z_duters@163.com (L.Z.); xiaocusuan@163.com (A.H.); fanwei@mail.dlut.edu.cn (F.M.); hnli@dlut.edu.cn (H.L.)

**Keywords:** bolt preload, digital image correlation, structural health monitoring, bolt looseness monitoring

## Abstract

Bolt connections are widely used in engineering structures but are susceptible to loosening during operation, which can result in significant safety concerns. Consequently, reliable bolt-loosening detection is of paramount importance. Conventional detection methodologies frequently exhibit deficiencies, including reduced efficiency, constrained accuracy, and the requirement for contact sensors. To overcome these limitations, this study proposes a novel non-contact approach for bolt preload monitoring based on Digital Image Correlation (DIC). In this method, an industrial camera captures speckle images of the bolt head before and after deformation, thereby enabling measurement of the surface strain. The DIC technique is employed to calculate the strain field on the bolt head surface, which exhibits a linear relationship with the bolt preload. The proposed method utilizes strain field tracking to facilitate effective and precise monitoring of bolt preload. Experimental results demonstrate that the method provides a precise, efficient, and user-friendly solution for bolt preload monitoring, showing great potential for applications in structural health monitoring.

## 1. Introduction

Bolted connections are utilized extensively in a variety of engineering and industrial applications due to their convenience and versatility. However, their integrity may be compromised by several factors, including dynamic loading [1], overloading, and corrosion [2]. The integrity of a bolted connection is contingent upon the bolt preload, which is instrumental in ensuring the reliability and safety of the associated equipment or structure. Insufficient preload has been demonstrated to accelerate bolt loosening and may ultimately lead to structural failure. Therefore, continuous monitoring of bolt preload is of paramount importance [3]. Over the past few years, scholars have conducted extensive and innovative research, proposing numerous techniques for monitoring bolt loosening [4]. These techniques can generally be classified into two categories: contact and non-contact methods. The distinction between these categories depends on the necessity of direct contact with the surface of the measured object during monitoring.

Common contact-based approaches include the tapping method, electromechanical impedance (EMI) method, and ultrasonic method [5]. The tapping method utilizes the fact that bolted structures emit distinct acoustic responses when struck. By analyzing the frequency characteristics of these sounds under different preload conditions, the state of the bolted connection can be inferred [6,7,8,9]. However, this method necessitates a testing environment that is devoid of noise, and it is highly susceptible to interference from environmental noise. This limitation compromises its accuracy in practical engineering applications. The EMI-based demonstrates consistent performance under diverse testing conditions and has emerged as a prevalent tool for damage assessment in various structures. This method involves attaching a piezoelectric sensor to the structure and evaluating the local health condition near the sensor [10,11,12,13]. The evaluation is achieved by measuring the coupled electromechanical impedance parameters of the structure and sensor under high-frequency sweep excitation. Nevertheless, one sensor is required for each bolt, making it difficult to assess the degree and location of loosening when multiple bolts are involved. The ultrasonic method can monitor the loosening of multiple bolts by attaching sensors to the connected structural components [14,15,16]. As ultrasonic waves propagate through the structure, their energy undergoes attenuation. Through meticulous analysis of this attenuation pattern, the condition of the bolt connection can be ascertained. Although this method has the capacity to can monitor multiple bolts simultaneously, its practical application is constrained by the elevated cost of sensors and associated equipment. In summary, the aforementioned bolt-loosening monitoring methods are contact-based and may introduce interference to the measured object during testing. Furthermore, the applicability of these methods is frequently constrained by environmental conditions, rendering them inadequate for specific bolt-monitoring scenarios.

The non-contact monitoring method eliminates the need to install sensors on the surface of the measured structure and imposes minimal constraints on the monitoring environment. In certain scenarios, non-contact approaches can effectively compensate for the limitations of traditional contact-based methods. Machine vision, often integrated with artificial intelligence (AI), has been widely adopted in non-contact monitoring and applied to bolt-loosening detection [17,18,19]. The general principle of this method is to compare digital images of bolts captured under different conditions to determine whether loosening has occurred. A commonly used vision-based technique involves quantifying the loosening angle. For instance, Park et al. [20,21,22] demonstrated a rotational-angle detection approach based on boundary detection and the Hough line transform. Similarly, Zhao et al. [18] proposed a deep learning–based method for calculating the loosening angle. Although the machine vision-based bolt-loosening monitoring is cost-effective and easy to implement, it lacks the sensitivity to detect early-stage bolt loosening and cannot accurately quantify bolt preload.

Digital Image Correlation (DIC) is an optical measurement technique that detects object deformation by analyzing digital images. Its extensive utilization in engineering practice is evident in its employment for measuring structural strain and deformation, as evidenced by numerous studies [23,24,25,26]. Chevalier et al. [27] employed DIC to analyze surface displacement fields of polymers under uniaxial and biaxial tensile loading, thereby obtaining non-uniform strain distributions of rubber-like materials under multi-axial conditions. Reder et al. [28] used laser irradiation to inscribe speckle patterns on the surfaces of ceramic and carbon fibers, enabling the acquisition of stress–strain curves in tensile experiments. Gencturk et al. [29] measured the full-scale deformation of prestressed concrete using DIC and compared the results with sensor-based measurements, thus validating the accuracy of DIC. These studies collectively demonstrated that DIC is a feasible, accurate, and highly sensitive technique capable of capturing even minute deformations. Jiménez Peña [30] further proposed a DIC-based approach for monitoring bolt preload by calculating bolt elongation. However, this approach is unsuitable for internal bolts or joints where the bolt head is embedded within the clamped material. Moreover, its measurement accuracy may be compromised for extended bolts due to a diminution in reduced spatial resolution.

In order to surmount the challenges of delayed detection, low accuracy, and high cost inherent in traditional bolt detection methods, this study proposes a Digital Image Correlation (DIC)-based approach for monitoring bolt preload. In this method, speckle patterns on the bolt surface are captured using an industrial camera before and after deformation. The acquired images are subsequently processed through DIC to compute the strain distribution on the bolt head surface. Consequently, experimental tests are performed to validate the effectiveness and accuracy of the proposed method.

## 2. Methodology

**Figure 1:**
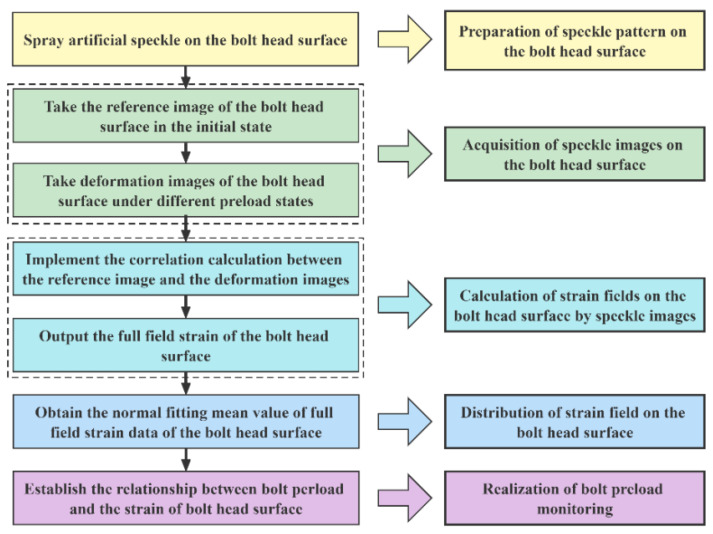
Basic implementation steps of the proposed method.

The methodology adopted in this study primarily consists of five stages: (1) preparation of a speckle pattern on the bolt head surface, (2) acquisition of speckle images, (3) calculation of strain fields, (4) analysis of the strain field distribution, and (5) estimation of bolt preload, as illustrated in Figure 1.

### 2.1. Preparation of Speckle Pattern on the Bolt Head Surface

For strain measurement using Digital Image Correlation (DIC), the quality of the speckle pattern on the bolt head surface is critical for accurately capturing deformation information. The standard approach for creating a speckle pattern typically involves manually applying matte black and white spray paint. To achieve a uniformly distributed pattern, a thin base coat of white primer is first sprayed onto the specimen surface. After the primer has solidified, a layer of black paint is randomly applied to form the speckle pattern.

**Figure 2:**
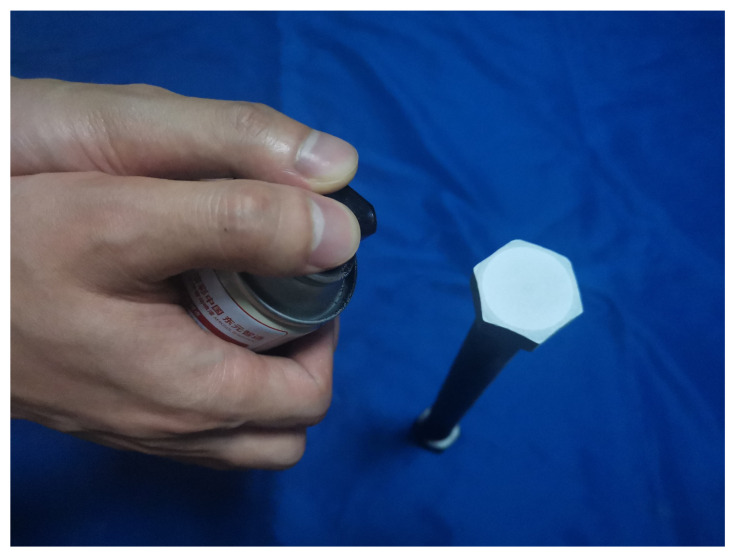
Method for spraying speckle patterns on the bolt head surface.

In this study, prior to testing, the bolt head surface was polished with sandpaper to remove burrs and ensure smoothness. Subsequently, matte paint was manually applied following the polishing process. During spraying, it is essential not to aim the spray gun directly at the bolt surface, as this may produce overly dense speckle clusters and introduce unwanted directional texture, thereby compromising the accuracy of the subsequent DIC analysis. Instead, the spray nozzle was positioned at an oblique angle relative to the bolt head surface, as shown in Figure 2, to ensure the formation of a uniformly distributed speckle pattern suitable for DIC measurement.

### 2.2. Computation of Strain Fields on the Bolt Head Surface Using Speckle Images

The Digital Image Correlation (DIC) method is an optical, non-contact technique for measuring displacement and strain. It provides full-field displacement and strain data by analyzing speckle patterns on the bolt surface captured in images taken before and after deformation, and by correlating the two images. The image acquired prior to deformation serves as the reference image, whereas the image captured after deformation is termed the deformed image.

**Figure 3:**
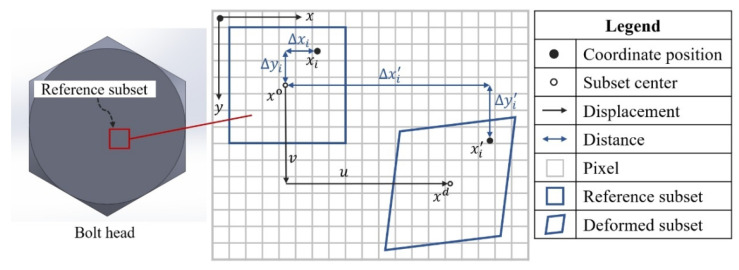
Schematic diagram of correlation calculation between reference subset and deformed subset.

The DIC algorithm determines the corresponding position of each subset in the deformed image by performing a cross-correlation analysis between the reference subset and the deformation subset, as illustrated in Figure 3. The successful matching of image subsets enables the calculation of displacement vectors and subsequent derivation of the strain field on the bolt head surface.

By using the coordinates of the center point $x_{0}$ in the reference subset, the coordinates of any point $x_{i}$ within the subset can be expressed as follows:

(1) $$\left[\begin{array}{c} x_{i}\\ y_{i} \end{array}\right]=\left[\begin{array}{c} x_{0}\\ y_{0} \end{array}\right]+\left[\begin{array}{c} \Delta x_{i}\\ \Delta y_{i} \end{array}\right]$$

where $x_{i}$ and $y_{i}$ denote the coordinates of the points in the reference subset along the *x* and *y* axes, respectively; $x_{0}$ and $y_{0}$ represent the coordinates of the center point in the reference subset along the *x* and *y* axes, respectively; and $\Delta x_{i}$ and $\Delta y_{i}$ indicate the coordinate offsets of the points relative to the center point along the *x* and *y* axes in the reference subset.

After deformation, the original point $x_{i}$ moves to a new location in the deformation subset, denoted as $x_{i}^{\prime }$, and its new coordinates can be expressed as:

(2) $$\left[\begin{array}{c} x_{i}^{\prime }\\ y_{i}^{\prime } \end{array}\right]=\left[\begin{array}{c} x_{0}\\ y_{0} \end{array}\right]+\left[\begin{array}{c} \Delta x_{i}^{\prime }\\ \Delta y_{i}^{\prime } \end{array}\right]$$

where $x_{i}^{\prime }$ and $y_{i}^{\prime }$ refer to the coordinates of the points in the deformation subset along the *x* and *y* axes, respectively; $\Delta x_{i}^{\prime }$ and $\Delta y_{i}^{\prime }$ represent the coordinate offsets of the points relative to the center of the reference subset along the *x* and *y* axes.

By calculating the values of $\Delta x_{i}^{\prime }$ and $\Delta y_{i}^{\prime }$ in Equation (2), the position of the reference subset within the deformed image can be determined. Owing to the non-rigid behavior of the bolts, the selected rectangular reference subset also undergoes deformation as the bolt preload changes. Therefore, a first-order shape function is utilized to describe the subset deformation, ensuring the accuracy of the subsequent subset-matching process, as illustrated in Equation (3).

(3) $$\left[\begin{array}{c} \Delta x_{i}^{\prime }\\ \Delta y_{i}^{\prime } \end{array}\right]=\left[\begin{array}{ccc} 1+u_{x} & u_{y} & u\\ v_{x} & 1+v_{y} & v \end{array}\right]\left[\begin{array}{c} \Delta x_{i}\\ \Delta y_{i}\\ 1 \end{array}\right]$$

where *u*, $u_{x}$, $u_{y}$, *v*, $v_{x}$ and $v_{y}$ are the parameters of the first-order shape function to be determined, and let $P={\left[u,u_{x},u_{y},v,v_{x},v_{y}\right]}^{T}$.

The two-dimensional digital image can be regarded as a sampling of light intensity, forming a matrix composed of the gray values of individual pixels. Let *F* and *G* denote the gray distribution functions of the reference subset and the deformed subset, respectively. Accordingly, the gray value at each pixel can be expressed as follows:

(4) $$\begin{array}{cc} f_{i} & =F(x_{i},y_{i}) \end{array}$$

(5) $$\begin{array}{cc} g_{i} & =G(x_{i}^{\prime },y_{i}^{\prime }) \end{array}$$

where $f_{i}$ and $g_{i}$ represent the gray values of individual pixels points in the reference subset and the deformed subset, respectively.

A correlation coefficient *C* is introduced to evaluate the similarity between corresponding points in the reference and deformed subsets. In this study, the zero-mean normalized cross-correlation (ZNCC) and zero-mean normalized sum of squared difference (ZNSSD) criteria are employed to perform the correlation analysis between the reference subset and the deformed subset,

(6) $$C_{\mathrm{ZNCC}}=\sum_{i=1}^{I}\frac{\left(f_{i}-\bar{f}\right)\left(g_{i}-\bar{g}\right)}{\tilde{f}\tilde{g}}$$

(7) $$C_{\mathrm{ZNSSD}}=\sum_{i=1}^{I}{\left[\frac{f_{i}-\bar{f}}{\tilde{f}}-\frac{g_{i}-\bar{g}}{\tilde{g}}\right]}^{2}$$

where *I* represents the number of pixels within the subset; $\bar{f}=\sum_{i=1}^{I}f_{i}/I$ and $\bar{g}=\sum_{i=1}^{I}g_{i}/I$ denote the average gray values of the reference subset and the deformed subset, respectively; and $\tilde{f}=\sqrt{\sum_{i=1}^{I}{\left(f_{i}-\bar{f}\right)}^{2}}$ and $\tilde{g}=\sqrt{\sum_{i=1}^{I}{\left(g_{i}-\bar{g}\right)}^{2}}$ are the normalized gray-value functions of the corresponding subsets.

Pan et al. [31] illustrated the relationship between these two correlation criteria as follows:

(8) $$C_{\mathrm{ZNCC}}=1-\frac{C_{\mathrm{ZNSSD}}}{2}$$

A first-order shape function is used to characterize the deformation of the reference subset, while the shape of the deformed subset is continuously optimized through nonlinear optimization during the correlation process [32,33]. When CZNCC approaches 1 or CZNSSD approaches 0, the optimized subset closely matches the actual deformation state of the deformed subset. Once the matching process is completed, the deformation information at the center point of the subset can be obtained. Several search algorithms have been developed to determine the location of corresponding subsets in the deformed bolt images, among which the Newton-Raphson (NR) algorithm is the most commonly employed [34]. The NR algorithm is utilized to iteratively compute the deformation vector, and the solution is obtained as follows:

(9) $$P=P_{0}-\frac{\nabla C(P_{0})}{\nabla \nabla C(P_{0})}$$

where $P_{0}$ denotes the initial value of the subset displacement vector; P represents the updated solution after each iteration; $\nabla C(P_{0})$ is the gradient of the correlation criterion; and $\nabla \nabla C(P_{0})$ is its second-order derivative, commonly referred to as the Hessian matrix.

The bicubic spline interpolation scheme is iteratively applied to determine the gray values at sub-pixel locations [35], as expressed in the following equation:

(10) $$g\left(x,y\right)=\sum_{m=0}^{3}\sum_{n=0}^{3}a_{mn}x^{m}y^{n}$$

where the coefficient $a_{mn}$ is determined by the gray values of the adjacent 4 × 4 pixel points centered on the interpolation point. The combination of NR algorithm with the bicubic spline interpolation scheme ensures high alignment accuracy and improved convergence stability. The sub-pixel displacement is obtained through the aforementioned iterative process. This procedure is repeated for multiple subsets to acquire the full-field displacement distribution of the component [36].

**Figure 4:**
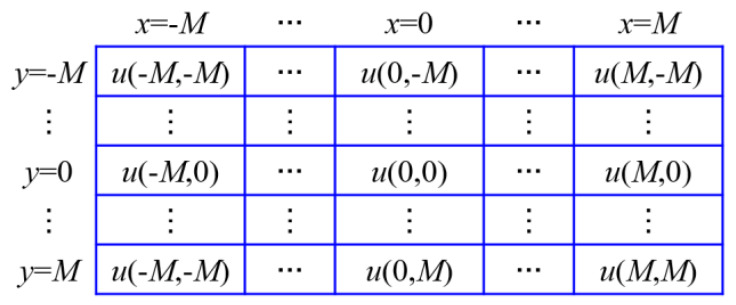
Displacement data for local least-squares fitting.

The full-field displacement data obtained from the previous calculations are utilized to compute the corresponding full-field strains through a specific strain evaluation method. Pan et al. [37] proposed a point-by-point local least squares fitting algorithm, which offers a practical and straightforward approach for strain computation with clear theoretical principles and simple implementation. To derive the full-field strains from the displacement field, a strain computation window with (2*M* + 1) × (2*M* + 1) data points is defined, with a specific data point selected as the widow center, as illustrated in Figure 4. The displacement data points within this window are then fitted using a two-dimensional polynomial, as expressed below:

(11) $$\begin{array}{cc} u(x,y) & =a_{0}+a_{1}x+a_{2}y\\ v(x,y) & =b_{0}+b_{1}x+b_{2}y \end{array}$$

where *x* and *y* range from −M to *M*; u(x,y) and v(x,y) denote the displacement field data in the *x* and *y* directions obtained from the DIC analysis; and $a_{i}$ and $b_{i}$ (where *i* = 0, 1, 2) are the polynomial coefficients to be determined.

The first equation in Equation (11) can be expressed in matrix form as follows.

(12) $$u=Xa\Rightarrow \left[\begin{array}{c} u(-M,-M)\\ u(-M+1,-M)\\ \vdots \\ u(0,0)\\ \vdots \\ u(M-1,M)\\ u(M,M) \end{array}\right]=\left[\begin{array}{ccc} 1 & -M & -M\\ 1 & -M+1 & -M\\ \vdots & \vdots & \vdots \\ 1 & 0 & 0\\ \vdots & \vdots & \vdots \\ 1 & M-1 & M\\ 1 & M & M \end{array}\right]\left(\begin{array}{c} a_{0}\\ a_{1}\\ a_{2} \end{array}\right)$$

By applying Equation (12), the coefficients can be obtained as $a={\left[\begin{array}{ccc} a_{0}, & a_{1}, & a_{2} \end{array}\right]}^{T}={\left(X^{T}X\right)}^{-1}X^{T}u$, where $a_{1}=\frac{\partial u}{\partial x}$ and $a_{2}=\frac{\partial u}{\partial y}$. Similarly, the coefficients $b_{0},b_{1}$ and $b_{2}$ can be determined, with $b_{1}=\frac{\partial v}{\partial x}$ and $b_{2}=\frac{\partial v}{\partial y}$. Consequently, Equation (13) yields the Lagrange strain component equations, in which $E_{xx}$ and $E_{yy}$ express the strain components in the *x* and *y* directions, respectively. This iterative procedure is performed point by point to obtain the full-field strain distribution.

(13) $$\begin{array}{c} \begin{array}{cc} E_{xx} & =\frac{1}{2}\left(2\frac{\partial u}{\partial x}+{\left(\frac{\partial u}{\partial x}\right)}^{2}+{\left(\frac{\partial v}{\partial x}\right)}^{2}\right)=\frac{1}{2}\left(2a_{1}+a_{1}^{2}+b_{1}^{2}\right)\\ E_{yy} & =\frac{1}{2}\left(2\frac{\partial v}{\partial y}+{\left(\frac{\partial u}{\partial y}\right)}^{2}+{\left(\frac{\partial v}{\partial y}\right)}^{2}\right)=\frac{1}{2}\left(2b_{2}+a_{2}^{2}+b_{2}^{2}\right) \end{array} \end{array}$$

## 3. Experimental Verification

To validate the feasibility and effectiveness of the proposed method, experiments were conducted using an M20 bolt, and the resulting data were analyzed. One critical factor influencing the measurement results is the selection of the region of interest (ROI) in the DIC analysis; therefore, this aspect was examined in detail. The experimental data obtained from the M20 bolt were used to evaluate the effect of different ROIs on the strain distribution of the bolt head surface. Furthermore, to confirm that the experimental results for the M20 bolt were not coincidental, additional verification experiments were carried out using bolts of different sizes—specifically, M18 and M22—to compare and analyze the DIC results across bolts with varying dimensions.

### 3.1. Instrumentation Setup

**Figure 5:**
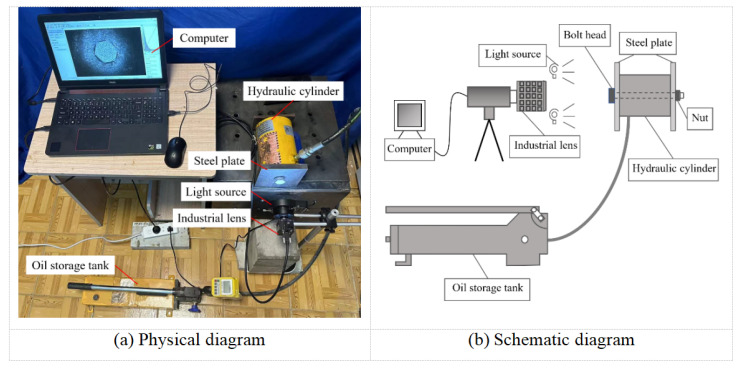
Experimental setup.

The experimental hardware primarily consisted of an industrial charge-coupled device (CCD) camera (6.3 MP; 3072 × 2048 resolution; Huagu Power WP-UC600, Shenzhen, China), a rock-bolt drawing dynamometer (0–300 kN; ML-300B; Cangzhou Zhaolong Zhongke Construction Equipment Co., Ltd., Cangzhou, China), a steel plate, a light source, a camera mount, and a laptop computer (Lenovo (Beijing) Co., Ltd., Beijing, China), as shown in Figure 5. To minimize interference from external illumination, the experimental setup was arranged in a low-light environment. A dedicated light source was used to ensure uniform illumination on the bolt-head surface, thereby enhancing image contrast and improving measurement accuracy by clearly defining the surface speckle patterns.

**Figure 6:**
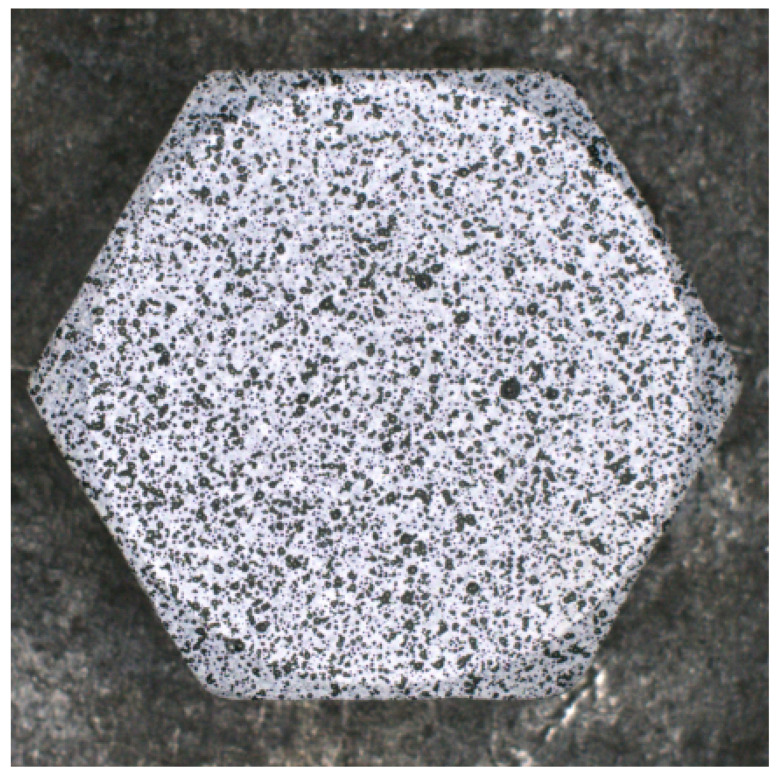
Surface of the M20 bolt head after preprocessing.

To establish the relationship between the bolt preload and the surface strain of the bolt head, a single-bolt axial loading experiment was conducted. Prior to testing, the surface of the M20 bolt head was preprocessed to generate a random speckle pattern, as illustrated in Figure 6. During the experiment, the hydraulic cylinder of the anchor puller was positioned horizontally on the workbench, and a square steel plate with pre-drilled holes was placed in front of it. The bolt screw was inserted through both the steel plate and the hydraulic cylinder, with the protruding end secured using an anchor tool. The bolt preload was applied by operating the handle of the anchor puller. To prevent bolt movement during the initial pre-tightening phase, the initial preload was set to 5 kN, after which the load was increased in increments of 1 kN. The total experimental preload range was thus set from 5 kN to 13 kN.

Subsequently, speckle images of the bolt head surface were captured at each 1 kN preload increment. Image acquisition was conducted by monitoring the liquid crystal display (LCD) while applying pressure to the handle of the anchor puller. The reference image (before deformation) and the deformed images (under various preload conditions) were then imported into the DIC analysis program to compute the strain field distribution on the bolt head surface corresponding to different preload levels.

### 3.2. Relationship Between Preload and Strain

**Figure 7:**
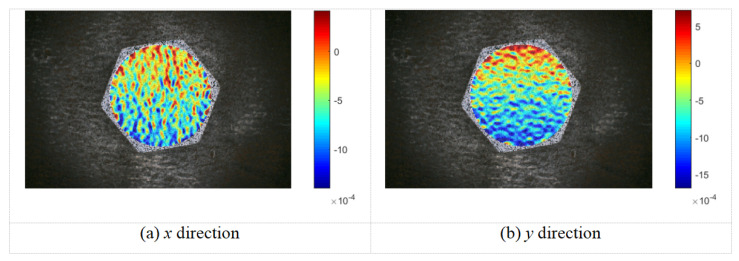
Strain field maps of the M20 bolt head surface in the *x* and *y* directions under a preload of 10.03 kN.

The M20 bolt was employed to verify the effectiveness of the proposed DIC-based method for monitoring bolt preload, with the experimental results analyzed according to the methodology described in the previous sections. Figure 7 presents the strain fields on the surface of the M20 bolt head in the *x* and *y* directions under a preload of 10.03 kN. Examination of the strain contour maps indicates that a relatively large number of pixels are concentrated near the middle of the color scale, while fewer pixels appear at both extremes. This distribution pattern is consistent with the bell-shaped profile of a normal distribution, suggesting that the measured strain field exhibits reasonable uniformity and stability.

As illustrated in Figure 7, the surface strain distribution on the bolt head is theoretically expected to exhibit symmetry. In practice, however, the distribution at individual points lacks a discernible pattern, as actual loading conditions often induce non-uniformity. Nevertheless, the measured strain field data indicates that, after normalization, the overall dataset aligns well with a normal distribution.

The strain field on the surface of the bolt head was obtained using the DIC technique, which provides strain data for each pixel within the analyzed region. To establish the correlation between the applied bolt preload and the resulting surface strain, a data processing strategy was developed. This strategy employs the strain field data corresponding to each specific preload condition to determine the representative strain value of the bolt under that load. The strain at each pixel of the bolt head surface varies with the applied preload. If a correlation exists between the surface strain field and the bolt preload, then a consistent relationship should also be observed between the average surface strain and the corresponding preload. To provide a more intuitive representation of the strain distribution, the average value derived from the normal fitting curve of the strain field data is used to establish this correlation. The probability density function of the normal distribution is expressed as follows:

(14) $$\begin{array}{c} f\left(x\right)=\frac{1}{\sqrt{2\pi }\sigma }\exp\left\{-\frac{{\left(x-\mu \right)}^{2}}{2\sigma^{2}}\right\} \end{array}$$

where f(x) represents the probability density function of the normal distribution, and σ and μ denote the standard deviation and mean value of the strain field data on the surface of the bolt head, respectively.

**Figure 8:**
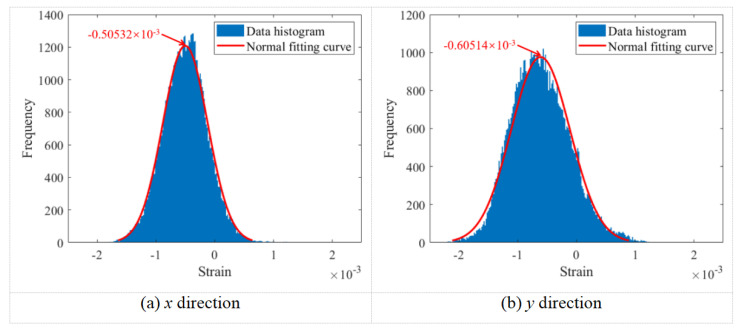
Normal fitting curves of strain field data in the *x* and *y* directions under a preload of 10.03 kN.

The relationship between the surface strain field of the bolt head and the bolt preload was determined by averaging the strain field data points across the bolt head surface. The normal fitting curves of the strain field data in the *x* and *y* directions under a preload of 10.03 kN are presented in Figure 8. The frequency on the *y*-axis refers to the number of speckle points sharing the same strain value; that is, it represents the count of occurrences for each specific strain magnitude among all analyzed points. As shown in the figure, the histogram of the strain field data on the bolt head surface closely follows a normal distribution pattern. To facilitate efficient subsequent data processing, a normal fitting was applied to the strain field data of the bolt head surface, and the mean value obtained from this fitting was used to establish the correlation between the bolt preload and the surface strain field.

**Figure 9:**
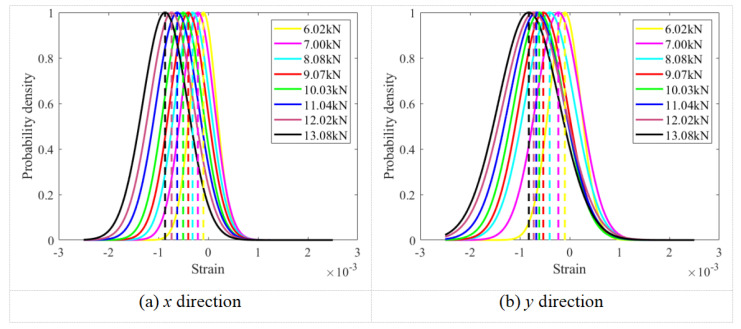
The change in the mean of the normal fitting for the strain field on the M20 bolt head surface in the *x* and *y* directions under different preloads.

Figure 9 presents the normal distribution fittings of the strain field data obtained from the surface of the M20 bolt head under various preload levels. The figure also shows the variations in the mean values derived from these fittings for the strain field data in the *x* and *y* directions. It can be observed that the mean strain values on the bolt head surface vary consistently with changes in the applied bolt preload.

**Figure 10:**
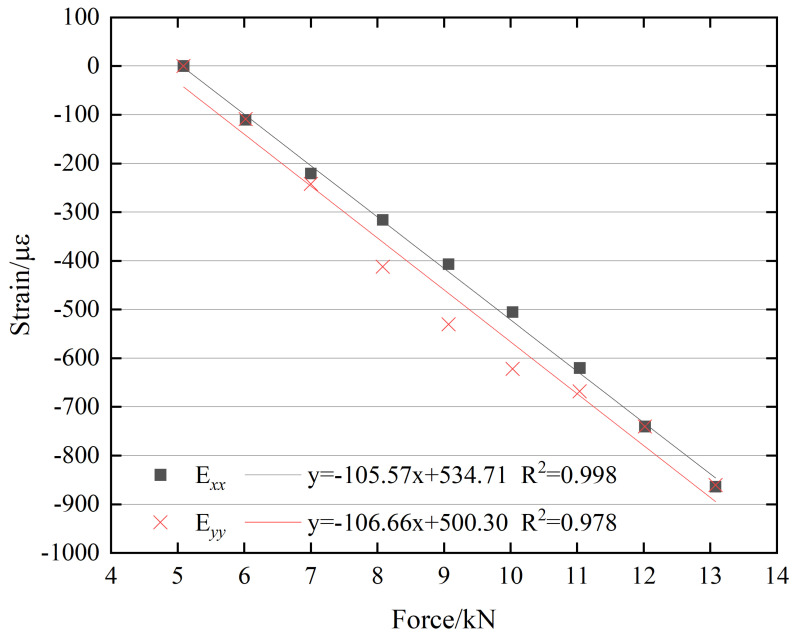
Linear relationship between bolt preload and surface strain of the M20 bolt head.

A linear regression analysis was conducted to establish the relationship between the bolt preload and the surface strain of the M20 bolt head, as shown in Figure 10. The figure indicates that the correlation coefficient of the regression equation is close to 1, demonstrating a strong linear relationship between the bolt preload and the mean surface strain. Overall, the relationship between the bolt preload and the surface strain on the M20 bolt head exhibits a clear linear increasing trend.

### 3.3. Influence of Region of Interest Selection

**Figure 11:**
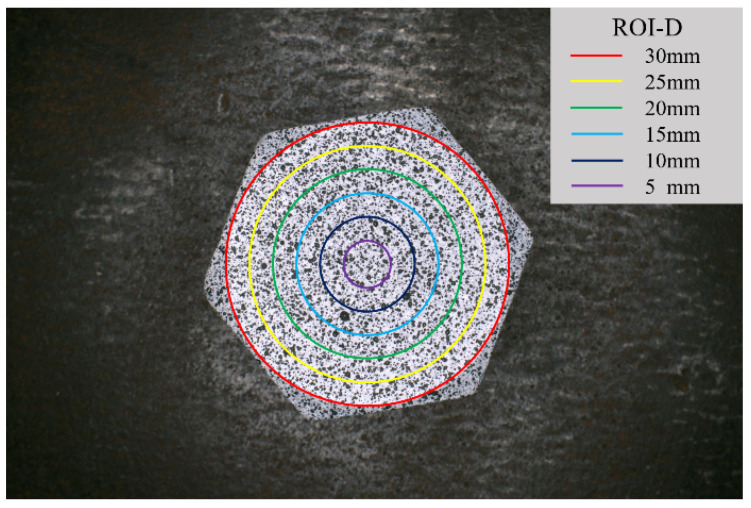
Regions of interest with different diameters on the surface of the bolt head.

Prior to conducting the DIC analysis, it is imperative to define the specific computational area—designated as the region of interest (ROI)—on the bolt head surface. In this study, experimental data obtained from the M20 bolt were used to examine the influence of ROI selection. The diameter of the M20 bolt head was 30 mm. The ROI on the bolt head surface was configured as a circular region, centered at the midpoint of the bolt head. The diameter of the circular ROI was varied from 30 mm to 5 mm, in increments of 5 mm, as illustrated in Figure 11.

**Figure 12:**
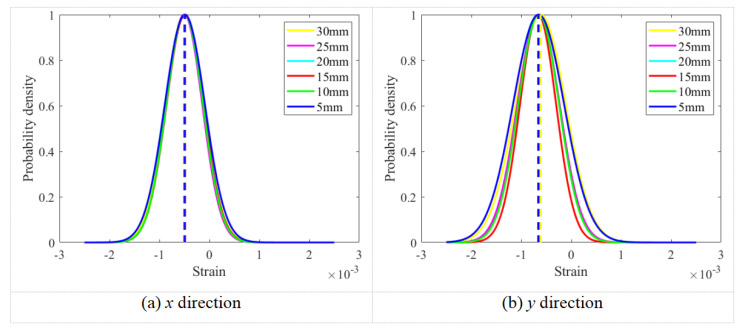
Normal fitting curves of the strain field data in regions of interest with different diameters in *x* and *y* directions under a preload of 10.03 kN.

The strain fields were computed using the experimental data obtained from six different ROIs, and normal fitting was applied to determine the mean strain values under each preload condition. The normal fitting curves of the strain field data within ROIs of varying diameters in the *x* and *y* directions under a preload of 10.03 kN are shown in Figure 12. As evident from the figure, the histograms of the strain field data corresponding to different ROIs on the bolt head surface in both the *x* and *y* directions follow a normal distribution, and the mean values obtained from the normal fittings in the same direction are approximately consistent.

The tested bolt preload range (6–13 kN) was divided into eight discrete preload levels, and the corresponding sequence numbers for each preload are listed in Table 1.

**Figure 13:**
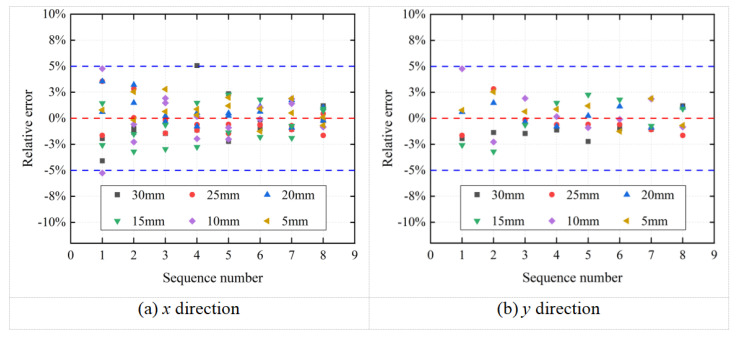
Relative error of bolt surface strain values under eight preload levels across six regions of interest in the *x* and *y* directions.

Figure 13 illustrates the relative error distribution of the surface strain values for the bolt under eight preload levels across six distinct ROIs in the *x* and *y* directions. As shown in the figure, the mean strain values obtained from different ROIs on the bolt surface under the same preload and in the same direction are nearly identical, with relative errors below 5%.

**Figure 14:**
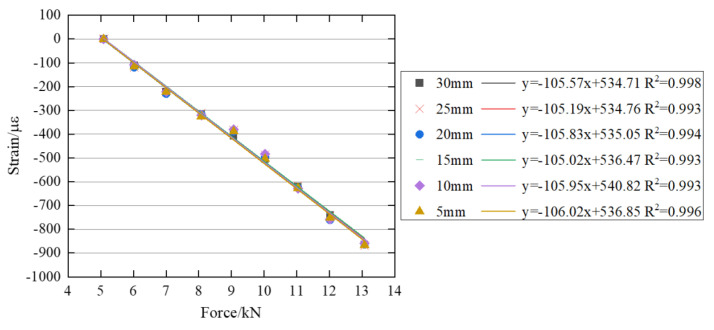
Relationship between bolt preload and surface strain of the bolt head in six regions of interest with different diameters in the *x* direction.

**Figure 15:**
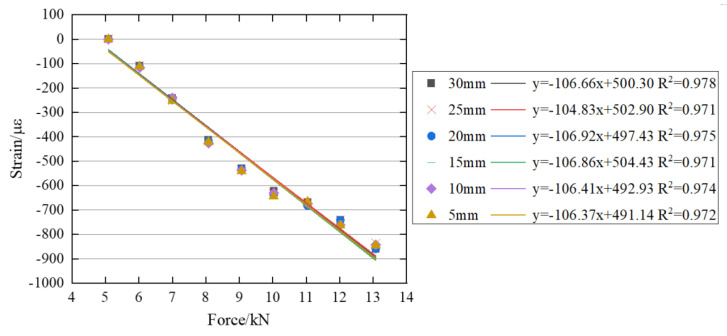
Relationship between bolt preload and surface strain of the bolt head in six regions of interest with different diameters in the *y* direction.

Figure 14 and Figure 15 illustrate the correlation between the bolt preload and the surface strain of the bolt head across six distinct ROIs with varying diameters in both the *x* and *y* directions. The experimental results confirm a linear increase in the surface strain of the bolt head with increasing bolt preload. Furthermore, the slopes of the linear fitting curves for different ROIs are nearly identical, indicating that the selection of ROI has minimal influence on the correlation between the bolt preload and the surface strain.

Experimental results suggest that choosing different ROIs does not notably impact the detected average strain value of the strain field on the surface of the bolt head. As a result, the relationship between the bolt preload and surface strain remains consistent regardless of the chosen ROI.

### 3.4. Verification Using Other Bolts Types

**Figure 16:**
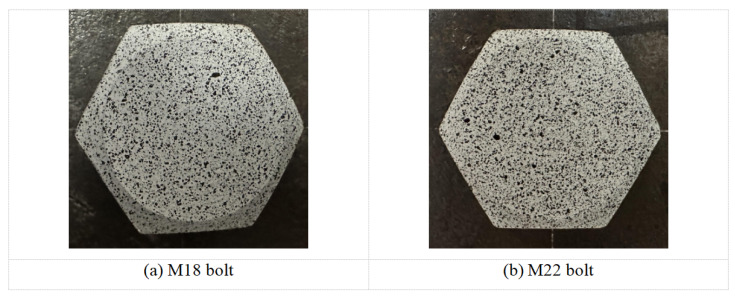
Speckle patterns on the bolt head surfaces of the M18 and M22 bolts.

To further validate the experimental results, similar tests were performed on M18 and M22 bolts. The speckle patterns on the surfaces of these bolts are shown in Figure 16. The speckle preparation procedure described in the earlier sections was adopted to generate the speckle patterns on the bolt surfaces.

**Figure 17:**
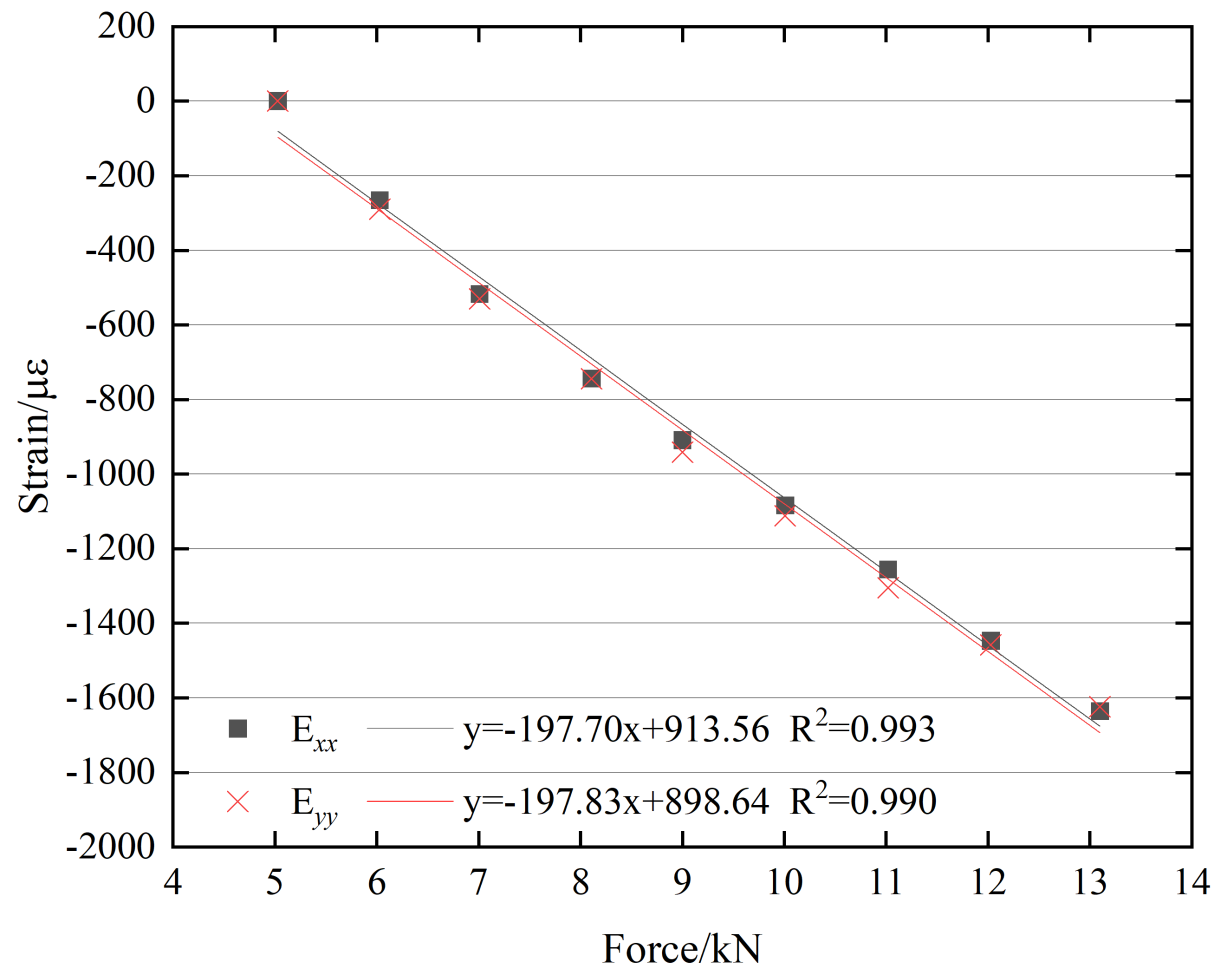
Relationship between the bolt preload and surface strain of the M18 bolt head.

**Figure 18:**
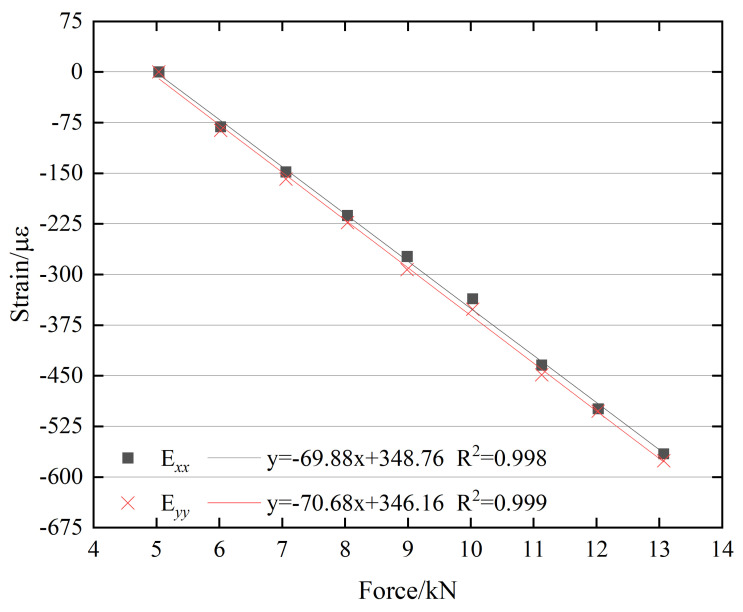
Relationship between the bolt preload and surface strain of the M22 bolt head.

The correlations between bolt preload and surface strain for the M18 and M22 bolts are presented in Figure 17 and Figure 18, respectively. The figures show that the correlation coefficients of the linear regression equations are close to 1, indicating a strong linear relationship between bolt preload and surface strain for both bolt types.

### 3.5. Correction of Bolt Rotation

**Figure 19:**
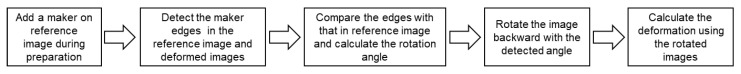
The flow chart of the rotation calibration.

To accurately address bolt deformation involving rotational movement, incorporating image rotation strategies during the pre-processing image registration phase is recommended. The specific workflow is as follows: During specimen preparation, geometric markers with distinct colors (e.g., rectangles or triangles) are placed on the bolt surface. For post-deformation images, integrating edge detection algorithms with color recognition technology yields optimal results for extracting the markers’ edge features. Subsequently, Principal Component Analysis (PCA) [38] is utilized to determine the principal orientation (slope) of the markers. Based on this, the rotation angle of the test image relative to the reference image is calculated. Finally, a reverse rotational transformation is applied to the test image using this angle. The corrected image is then suitable for the quantitative calculation of bolt deformation. The rotation calibration flowchart is shown in Figure 19.

**Figure 20:**
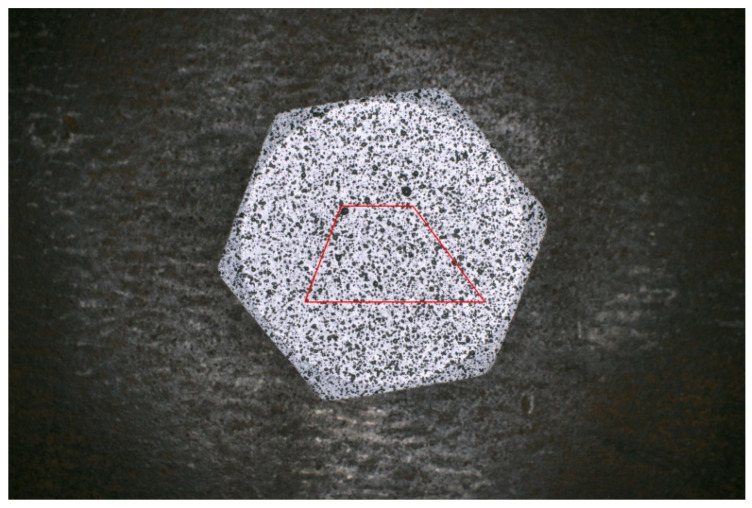
Selected M20 bolt image with trapezoid markings under a preload of 8.08 kN.

To validate the rotational correction method, an image of an M20 bolt was selected and trapezoidal markers were superimposed on the original image, as illustrated in Figure 20. The image was subjected to a series of rotations at angles of 0.8°, 1.7°, 3.4°, 5.0° and 6.1°, after which corrected images were generated using the proposed method. Subsequently, strain calculations were performed by comparing the pre-rotation and post-correction images. The measurement regions are shown in Figure 20, and the results are presented in Table 2.

Comparative experiments demonstrate that in the initial state without rotation, the measured strains in the *x* and *y* directions were −318μϵ and −420μϵ, respectively. Following the implementation of the rotational correction scheme proposed in this study, the maximum errors in the *x* and *y* directions were limited to 5 μϵ and 11 μϵ. These data confirm the validity and feasibility of the correction scheme, providing an effective solution for addressing measurement deviations caused by bolt rotation in practical applications. 

## 4. Discussion

While the correction algorithm proposed in this study effectively mitigates errors caused by in-plane rotation, we acknowledge that out-of-plane displacement remains a critical factor affecting measurement accuracy in a single-camera 2D-DIC setup. Specifically, any displacement along the optical axis alters the object distance, leading to variations in magnification. This optical phenomenon causes the DIC algorithm to misinterpret the change in image scale as tensile or compressive deformation, thereby introducing significant fictitious strains. Consequently, to overcome this inherent limitation, our future work will transition to a stereoscopic 3D-DIC system. By utilizing binocular vision principles to reconstruct the three-dimensional coordinates of the bolt surface, we can effectively decouple out-of-plane motion from the actual surface strain.

In future applications, it will be feasible to establish specific linear relationships between preload and strain for various bolt models. By comparing the DIC-measured strain data from in situ bolts against these pre-calibrated linear curves (derived from identical bolts under controlled conditions), bolt loosening can be effectively detected.

## 5. Conclusions

This paper proposes a novel digital image correlation (DIC)-based method for monitoring bolt preload, and its feasibility has been verified through experimental studies. The main conclusions are summarized as follows:

(1) As the bolt preload increases, the strain on the bolt head surface also increases. The strain field on the bolt head surface follows a normal distribution, and a strong linear correlation exists between the mean strain value and the bolt preload, with a correlation coefficient close to 1. These results demonstrate the feasibility of using DIC for quantitative bolt preload monitoring.

(2) The relative error of the strain values obtained from different regions of interest (ROIs) under the same preload is within 5%, and the slopes of the linear fitting curves representing the relationship between bolt preload and surface strain remain consistent across ROIs. This indicates that ROI selection has little influence on the accuracy of the proposed method.

(3) A strong linear correlation is also observed between the bolt preload and the surface strain for various bolt types (M18, M20, and M22), with regression equations yielding correlation coefficients close to 1.

Compared with traditional bolt-loosening monitoring methods, the proposed approach offers simple implementation, fewer constraints, and good applicability, showing strong potential for further development.

## Figures and Tables

**Table 1:** Sequence numbers corresponding to different preload levels.

Sequence Number	1	2	3	4	5	6	7	8
Preload (kN)	6.02	7.00	8.08	9.07	10.03	11.04	12.02	13.08

**Table 2:** Bolt rotation diagram and strain calculation results under a preload of 8.08 kN.

Rotation Angle	$0^{\circ }$	$0.8^{\circ }$	$1.7^{\circ }$	$3.4^{\circ }$	$5.0^{\circ }$	$6.1^{\circ }$
Before calibration	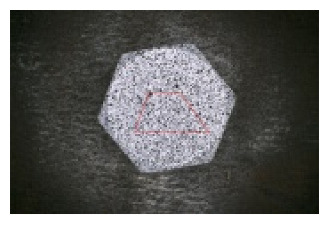	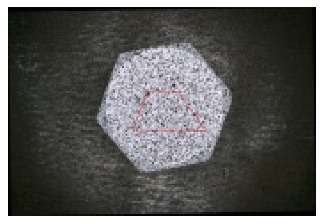	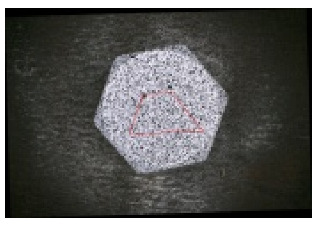	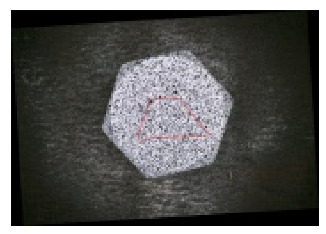	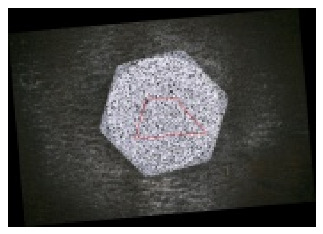	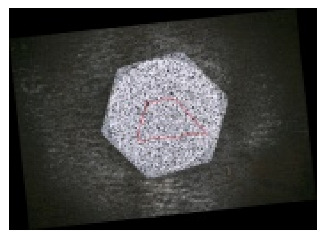
After calibration	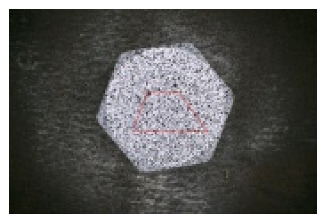	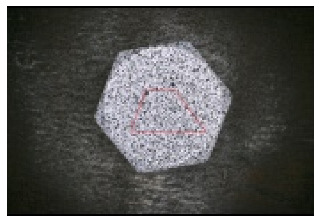	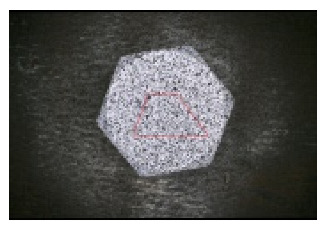	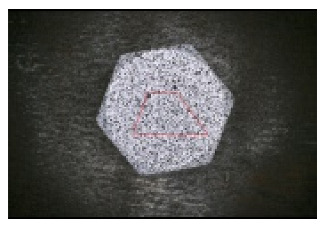	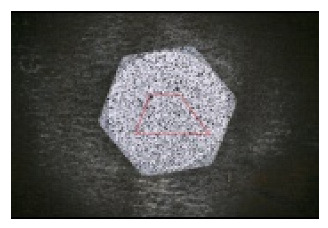	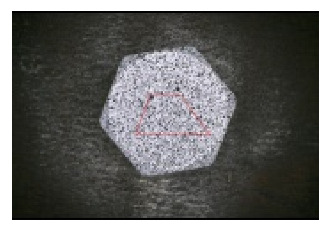
Average strain in the *x*-direction/μϵ	−318	−314	−315	−313	−315	−314
Average strain in the *y*-direction/μϵ	−420	−411	−409	−409	−410	−410

## Data Availability

The original contributions presented in this study are included in the article. Further inquiries can be directed to the corresponding author.
